# Incorporating the use of puppets and puppetry-based approaches in nutrition: a scoping review of interventions

**DOI:** 10.1017/jns.2026.10102

**Published:** 2026-05-12

**Authors:** Basil H. Aboul-Enein, Nada Benajiba, Majd Jan, Sarah Ali Alasmari, Patricia J. Kelly, Stephen F. Gambescia, Elizabeth Dodge

**Affiliations:** 1 University of Massachusetts Dartmouth, USA; 2 London School of Hygiene & Tropical Medicine Faculty of Public Health and Policy, UK; 3 Ibn Tofail University, Morocco; 4 Ibn Sina National College for Medical Studies, Saudi Arabia; 5 Saudi Electronic University, Saudi Arabia; 6 Thomas Jefferson University College of Nursing, USA; 7 Drexel University, USA; 8 College of Graduate & Professional Studies, University of New England, USA

**Keywords:** Education, intervention, nutrition, puppetry, puppets

## Abstract

Puppetry-based activities could serve as a low cost and manageable intervention in improving health-related outcomes. This review aims to identify the research to date and gaps in practice of the role and application of puppetry interventions in nutrition education. A scoping review was conducted using the PICOS framework and PRISMA-ScR guidelines, identifying studies across eight databases between January, 1980 and July, 2025.Twenty-five studies were identified, with the majority (*n* = 19) aimed at pre-school and school-aged children. Fifteen studies used quasi-experimental, pre–post designs. The use of hand and finger puppets was common, as well as videos and puppet theatre. Studies reported consistently positive findings in knowledge gain and psychosocial involvement, but mixed results on behaviour change. Little is known about the rationale for the type of puppet used, the scripts, and what audience was targeted for nutrition intervention messaging. Research could be undertaken to give more insight into matching what type of puppet to use to match the nature, scope, and extent of the educational message. Reports and recommendations in this review showed that participant engagement is a common and important objective. The gaps of knowledge in use of puppetry in nutrition education are many, thus creating opportunities for further evaluations and research, particularly in utilising what seems to be a manageable intervention within health promotion and disease prevention programmes. Puppetry can be a low cost, flexible, and easy to manage adjunct to nutrition education activities, providing culturally appropriate messaging with a range of audiences.

## Introduction

Puppets, or the moveable, material representation of human and/or animal characters with materials such as cloth, wood or clay, have been used to entertain children and adults since antiquity. Countries as diverse as Japan, India, Iran and Italy have a history of puppets manipulated with string, rods, hands, and shadows.^(1)^ Presentations can range from the highly professional shows designed to entertain, to educational presentations developed by teachers, librarians, and parents, giving useful information for healthy childhood development.^(2–5)^ For example, the long-running children’s TV show *Sesame Street*, popular worldwide, featured two puppets, Bert and Ernie, demonstrating while they had many differences, they could be good friends.^(6,7)^

The combination of puppetry as both theatre, art, and education has the potential to introduce discussion about sensitive topics, social barriers, and uninteresting but important topics that may be difficult with didactic education. Interaction and learning occur organically as observers identify with the different puppets and engage in implicit critical thinking. As an educational tool, puppets have been used around the world to directly and indirectly address a variety of health topics, including oral health in Peru,^(8)^ adolescent sexuality in India,^(9,10)^ suicide prevention in Canadian First Nations, ^(11)^ reducing the spread of HIV infection,^(12)^ drowning prevention in Spain, ^(13)^ and COVID-19 education with the elderly in China.^(14)^ Given the wide applicability and acceptability of puppetry and storytelling globally, puppetry based education is uniquely suited to cultural tailoring through the use of local puppeteers, and adaptation of scripts to local languages and cultural norms.^(2,5,11,12,15–17)^

Nutrition education is an essential cornerstone towards good health. Diets with adequate amounts of protein, complex carbohydrates, fats, and micronutrients can be a challenge to achieve with the bewildering variety of choices available in high-income countries, and the limited selections often available in low-to-middle income countries. Education for all ages provides the basis of the knowledge necessary for a balanced diet. Preschool and school age children can learn about fruits and vegetables, parents can implement strategies for maximising the purchase, preparation and offering of children’s healthy meals and snacks, and adults can learn about local resources. While children may not be responsible for the shopping and preparation of meals, they can be taught about healthy eating, about the importance of eating a variety of foods, and the role of nutrition in maintaining health.

For nutrition educators, cost is often a consideration when considering the development, implementation, and scalability of an intervention. Puppetry may be an attractive medium for educators to engage audiences through, both because of the ease of adoption of puppetry in education, particularly with the use of smaller and less complex puppets and sets, as well as the relatively low cost of implementation.^(1–5)^

Puppets hold appeal and attentiveness for both younger and older children, as well as adults given their range of characters from silly, surprising, sophisticated, to mysterious. Thus using them to teach about nutrition makes excellent pedagogical sense. Hand puppets, together with picture books, live demonstrations, and discussions are important strategies for providing children with the basics of nutrition knowledge. The aim of this review was to provide an inclusive description of the nature and extent and the role and application of puppetry interventions in nutrition education and their outcomes and recommendations.

## Methods

### Selection criteria

The Population, Intervention, Comparison, Outcomes, and Study (PICOS) design guidelines^(18)^ were incorporated to develop the research question: *‘*‘Do adults and children populations of all age groups’ (P) that are offered puppetry-based interventions (I) have improved nutrition-related outcomes (O) compared with those that do not participate in puppetry-based interventions(C)?’ and subsequent inclusion and exclusion criteria (see Table 1). Peer-reviewed articles published in the English language were included. Interventions reported outside traditional peer-reviewed articles were excluded in this review. The search was conducted in the summer of 2025, and the results communicate the literature published between 1980 and July 2025, given a robust longitudinal review of the state of puppetry assisted interventions in nutrition education.

**Table 1.** PICOS criteria for inclusion and exclusion of studies

Parameter	Inclusion criteria	Exclusion criteria
**Population**	Adults >18years children and young people aged 0-17years	N/A
**Intervention type**	Any form of puppetry-based intervention that address nutrition education/behaviour-related outcomes	Interventions that are not based on the use of puppetry. Interventions that do not address nutrition education/behaviour-related outcomes.
**Comparators**	Pre-intervention, baseline variables (i.e. knowledge, attitudes, beliefs etc.) of groups who were: Control: received no intervention. Received partial intervention (e.g. educational intervention only or multi-componential intervention.)	N/A
**Outcomes of interest**	Changes in nutrition behaviour-related outcomes Changes in nutrition-related knowledge, beliefs, attitudes etc.	Non-nutrition related outcomes
**Language**	English, Spanish, Portuguese, or French	All other languages
**Study Type**	Intervention studies with quantitative, qualitative, experiential, or observed outcomes. Peer-reviewed original research articles Original research conference publications	Non-numeric/categorical assessments or qualitative studies Non-Peer-reviewed articles Study protocols Commentaires Narratives Communications Non-intervention based studies White papers Similar article types Grey literature

### Search strategy

**Figure 1.**
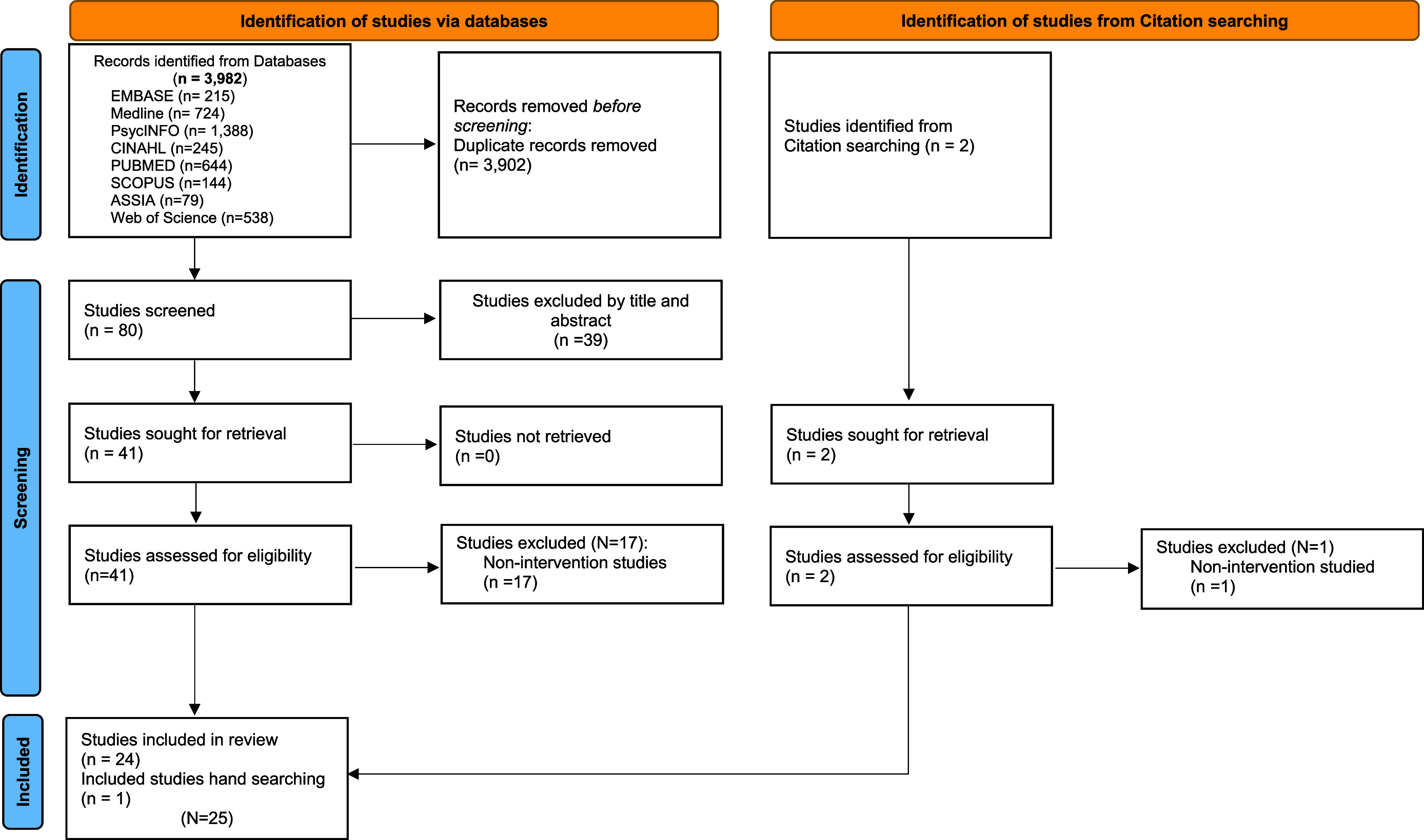
2020 PRISMA scR flow diagram.

This review used the PRISMA-ScR extension for scoping review guidelines; there was no pre-registration or protocol submitted.^(19)^ A search was conducted using eight academic databases and a combination of subject heading keywords, terms, phrases, and Boolean operators were used (see Table 2). Search strategies were adapted in accordance with the indexing systems of each respective database used (see supplemental material). Two of the authors [B.A.E. and N.B.] conducted the searches for relevant articles. Author [B.A.E] utilised Rayyan QCRI software^(20)^ to assist in the screening process. All retrieved articles were screened for relevance to the topic. In addition, reference lists from retrieved articles were also manually searched to identify any additional publications that satisfied the eligibility criteria. Titles and abstracts were screened for relevancy, and potentially relevant journal abstracts were reviewed by two authors [N.B. and B.A.E.]. Potential articles for inclusion in this review were evaluated independently for relevance, merit, and inclusion/exclusion criteria. Articles accepted for inclusion were individually reviewed by each author. Any disagreement was resolved by consensus. Figure 1 provides the PRISMA flowchart leading to selected articles for this review (see Figure 1). We explored the characteristics of interventions, target audiences, and outcomes and tabulated the included studies. Given that methodological quality assessment is not a prerequisite for scoping reviews, we did not appraise the included studies.^(21)^

**Table 2.** Electronic databases used with relevant search period and terms

Databases	Search period	MeSH keywords, terms, phrases, and Boolean operators
EMBASE, Medline, PsycINFO, CINAHL, PUBMED, SCOPUS, ASSIA, and Web of Science	January 1^st^ 1980 to July 31^st^ 2025	‘Nutrition’ [MeSH] OR ‘Diet*’ [MeSH] AND ‘Intervention’ [MeSH]; OR ‘Program’ [MeSH]; OR ‘education’ [MeSH]; OR ‘promotion’ [MeSH] AND ‘Puppets’ [MeSH]; OR ‘puppetry’ [MeSH]; OR ‘puppeteer’ [MeSH]; OR ‘puppet show’ [MeSH]; OR ‘marionette’ [MeSH]; OR ‘ventriloquy’ [MeSH]; OR ‘ventriloquism’ [MeSH]

## Results

A total of 25 intervention studies (see Table 3) that met the eligibility criteria was included in this review. The search dates applied were from January 1, 1980 through July 31, 2025, and the reviewed studies were published between 1979,^(22)^ and 2025^(23)^ with slightly less than half of the publications (*n* = 10) during the 2010s.^(24–33)^ The research studies were conducted in 13 different countries, with six in Indonesia,^(33–38)^ seven in the USA,^(24,25,28,39–42)^ two in the Netherlands,^(26,29)^ and one in each of the following countries – Brazil, India, Colombia, the Philippines, Bolivia, Spain, France, Egypt, and Ecuador.^(9,22,23,27,30–32,43,44)^

**Table 3.** Summary of literature search (*N* = 25)

Author, year; country	Target population/ sample	Type of study	Type and details of Intervention	Measured parameters	Main results
Abdelrahman et al. (2025); Egypt	91 children, 6–12 years old	Quasi-experimental, pre-post-test	Two-arm intervention with control group covering the healthy eating pyramid, healthy nutrition, food groups, illustration of good eating habits and examples of good food choices; the control group received a 20-25 minute lecture with group discussion, flashcards were used in the first intervention group, in addition to the lecture and discussion, and the second intervention group participated in a 20-25 minute scripted puppet show covering the same concepts	Knowledge, attitudes	Significant improvement in knowledge from pre- post-test assessments. Post-test, the puppet show group had the highest score (*p* = 0.00). The differences in pre- post-test scores were significant for all groups (P < 0.05). Significant increase in attitudes towards healthy nutrition from pre- post-test. Post-test, the puppet show group had the highest score (*p* = 0.002). The differences in pre- post-test scores were significant for all groups (P < 0.05)
Alessi et al. (2014); Brazil	202 children, 2.5–6 years old	Mixed methods	Age-specific activities (puppet theatre, sensory exploration, ‘my plate’ crafts) to encourage fruit and vegetable acceptance	Acceptance of functional foods in school meals	Post-intervention acceptance of functional foods increased 9 %
Anggraini et al. (2021); Indonesia	48 students 4–5 years old	One group pre–post test	4-session programme used puppet shows, storybooks, hands-on activities (puzzles, cards) to promote fruit and vegetable consumption in toddlers	Fruit and vegetable consumption, toddler behaviour	Significant improvement in fruit & vegetable consumption post intervention (*P* = 0.000)
Arora et al. (1980); India	195 primary school students 9–12 years old	One group pre–post test	School-based nutrition lecture was delivered, followed by one of nine different reinforcement methods, e.g., blackboard notes, storytelling, puppets	Knowledge	Puppet used as a method of education showed significant changes in nutrition knowledge (p-<0.001
Bertrand and Bertrand (1979); Colombia	100 adult women, 15–49 years old	Two group pre-post-test	8-month community health programme (*Vivamos Mejor*) of education with talks, films, puppetry, home visits on health and nutrition	Knowledge, awareness, behaviour	No significant difference in nutrition knowledge between groups
Bevelander et al. (2013); Netherlands	141 children, 6–8 years old	RCT	Lesson on peer modelling; one group with puppet present as a visual cue.	Weight, food intake, test taste	No significant effect of the animated intervention on consumption. (*P* = 0.34)
Byrd-Bredbenner et al. (1993); USA	1000 Head Start programme children	Two group pre–post test	6-week nutrition curriculum for preschoolers of puppets, games, stories; teacher training to implement programme	Knowledge, attitudes	No significant change in knowledge between groups; significant differences in attitudes for ‘I like to eat nutritious food; new food’.
Darawati et al. (2020); Indonesia	66 mothers & children, 2–5 years, with stunting	Two group pre–post test	Compared nutrition counselling with puppets for mothers of stunted children to conventional counselling	Knowledge, nutrient intake	Significant increase in knowledge and consumption of energy & nutrients in treatment compared to control group (*p* < 0.05)
de Droog et al. (2017); Netherlands	163 toddlers 2–3 years old	Intervention study	A study testing interactive vs. passive story reading, with or without a puppet, over four days, followed by an assessment of toddlers’ vegetable consumption.	Carrot consumption	No significant effect of the puppet show on carrot consumption.
Fitzgibbon et al. (2006); USA	400 preschool children	RCT	14-week bilingual intervention with 20-minute 3x/week sessions of puppet-based nutrition education, 20 minutes of PA	BMI, dietary intake physical activity	No significant increase in BMI between the intervention & control group after 1 year; no difference in dietary intake, exercise frequency
Fitzgibbon et al. (2013); USA	157 preschool children 3–5 years	Pilot study	A 14-week, culturally tailored family intervention included child sessions (puppet-led nutrition, exercise) and parent classes, compared to a general health control group.	Anthropometric measures. Physical activity. Dietary intake Foods consumed outside of preschool. Meal observation at school.	Significant differences between groups for BMI changes (p-value = 0.003)
Glorioso et al. (2018); Philippines	1857 students, 10 –17 years old	One-group pre–post test	5-minute educational puppet video about diabetes and physical activity	Knowledge	Significant increase in knowledge scores (*p* = 0.000)
Goodwin et al. (2015); USA	12 children, 3–5 years old	One group pre–post test	6-week puppet-based nutrition education programme	Macronutrient intake	Significant decrease in calorie intake (*p* < 0.05); no decrease in total & simple carbohydrate, fats
Kumbhar (2023); India	100 5^th^ and 6^th^ graders and 20 teachers	One group pre–post test	One school-based puppet show on junk food	Student knowledge about health impacts of junk foods, teacher perception about puppet show as a pedagogical method	Significant increase in knowledge scores of health effects of junk foods (*p* = 0.01), teacher perception of puppetry as pedagogical method increased
Martinez et al. (2018); Bolivia	2000 pregnant women, children<12 months old	RCT	Monthly home visits with play-based methods (puppets, songs, theatre) of nutrition hygiene messages to caregivers	Knowledge, feeding practices, food spending, diet diversity	No significant changes in anthropometric or anaemia
Murdani et al. (2021); Indonesia	64 Kindergarteners	One group pre–post test	One week of puppet-based games about increasing fruit and vegetable consumption	Fruit and vegetable consumption	Significant increase (*p* = 0.000) in fruit and vegetable consumption in the intervention compared to control group
Nicklas et al. (2017); USA	253 preschoolers, 4–5 years old	Randomised feasibility intervention with control	Four-week intervention with 4 puppet shows, 1 shown each day for a consecutive week before lunch and also sent home for use at least once during the intervention week	Fruit and vegetable dish consumption	Significantly (p < 0.0001) increased consumption of vegetable dishes in the intervention compared to the control group. At follow-up, intervention group retained significantly (p = 0.022) higher intake of vegetable dishes compared to the control group
Perez-Rodrigo and Aranceta (1997); Spain	150 public school students, 8–12 years old	One group pre-post-test, mixed method	Year-long, school programme combining classroom education (games, puppets), cooking workshops, school lunch changes	Knowledge, cooking, hygiene	Significant changes in knowledge, skills, eating, except for candy (*p* < 0.001)
Pélicand et al. (2006); France	14 children with diabetes, 10–12 years old	Two group pre–post test	Therapeutic camp for diabetic children; one group used self-made puppets for emotional expression, control group standard discussions	Emotional expression, diabetes attitudes, behaviours, engagement	Puppets facilitated deeper emotional expression and more constructive attitudes.
Permanasari et al. (2022); Indonesia	25 preschool children	One-group pre–post test	3-weekly storytelling sessions using finger puppets to convey healthy eating messages to preschoolers	Knowledge	Significant improvement in nutrition knowledge
Romo and Abril-Ulloa (2018); Ecuador	155 children, 3–4 years old.	One group pre–post test	3–7-month intervention of puppets, characters, songs to promote water, fruit/vegetable intake & physical activity	BMI, consumption of water, sugar-sweetened beverages, fruits, vegetables for snacks, screen time	Significant pre–post intervention improvements in mean BMI- (*P* = .002), sweetened beverage & fruit/ vegetable intake (*P* < .001), screen time (*P* = .03).
Setiana et al. (2020); Indonesia	64 preschool children 5–6 years old	Three group pre–post test	Storytelling (using finger puppets) & flashcards compared to lecture for improving preschoolers’ nutritional knowledge	Knowledge	Significant knowledge improvement in treatment compared to control groups (*p* < 0.001); no difference between flash cards & storytelling methods
Sharma et al. (2011); USA	75 children, 3–5 years old	One group pre–post test	6-week pilot *CATCH Early Childhood Program*, 4 parts: teacher-led puppet-based nutrition curriculum, PA, parent education, teacher training	Dietary intake, physical activity	No significant increase in fruit juice and vegetable consumption
Usfar et al. (2020); Indonesia	32 preschool teachers	One group, pre–post	15-month intervention. Teachers trained on nutrition, child development, given educational aids of finger puppet, storybooks	Knowledge	Teachers retrained increased knowledge after 15 months
Wright et al. (2007); USA	55 children, grades 1–3	One group, pre–post	One 30-minute puppet show	Knowledge about diet and physical activity	Increases in student confidence to achieve their health goals and in knowledge that healthful food and exercise is important were demonstrated post-intervention

BMI, Body mass index; PA, physical activity; RCT, randomised control trial.

### Sample sizes and participant characteristics

The target populations varied and included different age groups. Most interventions (*n* = 19) were aimed at preschool and school-aged children.^(23–28,31,33,35–45)^ Other studies concentrated on specific subgroups, including toddlers,^(27,29)^ diabetic children,^(9)^ adolescents,^(31)^ mothers of stunted children,^(36)^ of children with stunting (short height for age), preschool teachers,^(34)^ pregnant women,^(32)^ and women of reproductive age.^(22)^ Sample sizes varied widely, ranging from small pilot studies (e.g., *n* = 12, *n* = 14) to larger trials (e.g., *n* = 2000 households, *n* = 1857 students).

#### Type of studies and details intervention

The systematic review includes various study designs that assess interventions. Most of the studies (*n* = 15) were quasi-experimental pre–post interventions.^(22,23,28–31,33,35–39,42,43,45)^ The review included several randomised controlled trials,^(26,32,40,41)^ multiple pilot studies,^(24,25,34,44)^ and one mixed-methods study.^(27)^

Overall, the interventions employed in the included studies differed in their delivery, duration, and specific components. The length of interventions in the review had a wide range, from a single brief exposure to programmes with puppet content to those that spanned several years. The shortest interventions included a single lesson^(26,42,45)^ and a 5-minute video.^(31)^ Others had content that lasted for several days or weeks. Common durations were 6 weeks^(24,28,39)^ and 14 weeks.^(25,40)^ Some studies carried out interventions over several months, such as 3–7 months,^(30)^ 8 months,^(22)^ and monthly visits.^(32)^ The longest intervention reported lasted one year.^(44)^ A few studies gave the number of sessions instead of a timeframe, noting three sessions,^(35)^ four sessions,^(37)^ and a summer camp format.^(9)^

The use of puppetry was diverse and was done in various ways across studies, such as hand puppets for nutrition lessons,^(25,40)^ finger puppets for storytelling,^(33,35)^ puppet theatre,^(27)^ puppet videos,^(31)^ and as a tool in nutrition or therapeutic counselling.^(9,36)^ Interventions were held in various settings, including schools,^(26,27,39,43,44)^ community centres,^(22)^ summer camps,^(9)^ and household.^(32)^ Many studies combined puppet-based education with other strategies, such as physical activity,^(30,40)^ cooking workshops,^(44)^ parent education,^(24,25)^ and environmental changes like menu modifications.^(44)^

### Measured parameters

The parameters measured in the systematic review are varied. They included outcomes based only on knowledge,^(31,33–35,43)^ assessments that combined knowledge, attitude, and behaviour,^(9,22,32, 39, 44)^ and specific dietary and consumption measures.^(24,26–29,37)^ Some studies focused on anthropometric and health outcomes, including body mass index (BMI), physical activity, and dietary intake details.^(25,30,40)^ Other studies evaluated metrics related to caregivers, such as maternal knowledge, feeding practices, and household nutrient intake.^(32,36)^

### Knowledge and psychosocial outcomes

The most consistent positive findings appeared in studies measuring knowledge gain and psychosocial involvement. Multiple studies showed significant improvements in nutrition knowledge after puppet-based interventions.^(23,31,33,35,36,42–45)^ Additionally, Pélicand *et al.* ^(9)^ found that puppets helped children with diabetes express emotions better and develop more positive attitudes. Byrd-Bredbenner *et al.* ^(39)^ noted modest improvements in specific food attitudes. On the other hand, some studies did not find any significant change in knowledge,^(22,28,39)^ indicating that knowledge gains are not guaranteed and might rely on other factors in implementation such as peer attitudes.

### Behavioural and dietary outcomes

The results for actual behaviour and dietary intake showed more variation with several studies reporting positive results. For instance, Romo and Abril-Ulloa^(30)^ found a significant reduction in the consumption of sugar-sweetened beverages and a reduction in screen time. Darawati *et al.* ^(36)^ noted better nutrient intake among mothers, and three studies observed increased fruit and vegetable consumption in children.^(37,38,41)^ Alessi *et al.* ^(27)^ also reported greater acceptance of functional foods. However, other studies found no significant changes in dietary intake,^(24,40)^ vegetable consumption,^(29)^ or candy consumption.^(26)^ Goodwin *et al.* ^(28)^ showed a notable decrease in the calories selected by children but no change in macronutrient composition or their ability to identify high-fibre foods.

### Anthropometric and health outcomes

Evidence for the impact on physical health measures was inconsistent. Fitzgibbon *et al.* ^(25)^ found a significant difference in BMI changes between groups. This result was backed by Romo and Abril-Ulloa,^(30)^ who reported significant improvements in BMI-for-age. In contrast, other studies found no significant effects on BMI,^(40)^ anthropometry, or anaemia rates.^(32)^

### Main recommendations from included studies

The main recommendations from the studies highlighted the need for careful evaluation of health education programmes.^(22)^ They also stressed the importance of developing engaging materials that are suitable for different age groups.^(37,39,43)^ Interdisciplinary collaboration across different settings, especially between schools and parents, was indicated as crucial.^(33,44)^ Several studies recommended using puppets as an effective tool in both therapeutic and educational environments.^(9,35,36)^ A recurring theme is the need for culturally tailored family-centred interventions.^(25,40)^ Additional suggestions included incorporating specific topics into school curricula^(31)^ and menus,^(27)^ and designing programmes that consider strong social influences, like peer modelling.^(26)^ In terms of research, recommendations indicated a focus on long-term effectiveness, sustainability, and cost-effectiveness were proposed.^(24,30,32)^

## Discussion

The existing body of nutrition education programmes incorporating puppetry remains limited, with only 25 evaluated interventions over nearly 45 years identified for the purposes of this scoping review. Nonetheless, these studies span 13 countries, multiple age groups, and a range of settings, indicating that puppetry has been applied across diverse educational, nutrition-focused and cultural contexts. Across the studies, puppetry-based interventions most consistently improved nutrition knowledge,^(23,33–36,42–45)^ however, several studies did not detect significant knowledge gains.^(22,32,39)^ Pélicand *et al.* ^(9)^ used puppets to facilitate emotional expression among children with diabetes, highlighting how puppetry can support psychosocial skills alongside content learning. The overall heterogeneity in design and measurement means that improvements in knowledge are promising but not yet definitive.

Behavioural, anthropometric, and dietary outcomes were more mixed. Several studies found intervention impacts on anthropometrics,^(25,30)^ fruit and vegetable intake,^(37,38,41)^ acceptance of functional foods,^(27)^ and improvements in macronutrient intake,^(28)^ while others did not have comparable findings on similar metrics such as BMI and weight,^(25,26)^ and dietary intake.^(24,29)^ While these findings align with broader evidence that multifaceted nutrition education and physical activity interventions can influence weight trajectories,^(46–49)^ some of the reviewed puppet-based interventions were not powered or designed to detect long-term anthropometric effects.^(25,30,32,40)^

A key theme across puppetry-based interventions included in this review is the importance of active engagement and the applications of social learning. Interventions that include experiential components such as taste testing, role-play, or interacting with characters appear particularly effective. Both puppetry-based educational videos and live shows improved knowledge or nutrient intake through framing abstract health messages and concepts in ways that the audience found relatable and emotionally resonant.^(9,23,24,27,31,36,37,41,43,45)^ However, few studies reported specifically how puppet characters were developed, scripted, or adapted to reflect participants’ culture, identities, languages, or social environments,^(24,27,29,30–35,37,38,45)^ even though arts-based health literature suggests that culturally grounded characters and narratives can strengthen identification and message relevance.^(2–5,11,12,17,36)^ Incorporation of additional methodological information in future interventions on puppet type, character design, and scripting decisions would help further elucidate how these elements influence both audience engagement and intended intervention outcomes.

Another educational approach across the studies reviewed is the frequent use of puppetry to introduce or reinforce nutrition information or nutrition related behaviour combined with other multi-component aspects in support of the intervention (such as teacher- or parent-led activities, take-home materials, or environmental modifications) intended to support behaviour change.^(23–25,27,30,32,34,36–38,41,44)^ This aligns with broader behaviour-change models that emphasise layering information, skills practice, social support, and structural changes to influence diet-related behaviours.^(50–54)^ Within this framework, puppetry-based interventions may be particularly well suited for capturing attention and engaging the audience, modelling dialogue about complex topics and concepts, and lowering barriers to participation across diverse audiences and participant age groups; however, additional intervention components are needed to sustain and translate outcomes from puppetry-based interventions into everyday food choices.

The review highlights important gaps in the current evidence base. First, there is limited use of standardised, validated measures of dietary intake, psychosocial constructs, and anthropometric outcomes, which complicates cross-study comparison. Second, most interventions in this review assessed outcomes immediately or shortly after the puppetry-based intervention, with relatively few studies incorporating longer-term follow-up. Therefore, it remains uncertain whether observed gains in knowledge or short-term changes in diet-related behaviour are sustained over time. Third, almost none of the included interventions utilised a control group or explicitly compared different puppet formats, story styles, or degrees of audience participation, even though emerging applied-puppetry and arts-based health research suggests that these design choices may influence engagement and effectiveness

Future research should therefore prioritise more rigorous study designs, including randomised or well-controlled quasi-experimental trials; transparent methodological designs describing puppet design, script development, and intervention implementation processes; and incorporation of longer follow-up timeframes to capture maintenance of behaviour change. Studies that explicitly test puppetry as a standalone component as compared to puppetry-based interventions that are developed in combination with other educational strategies would help to determine whether puppets primarily act as a tool to capture the interest of and engage the audience, or whether puppetry can be utilised in nutrition education intervention settings to facilitate positive impacts on nutrition- and health-related knowledge, attitude, and behaviour outcomes.

Finally, this review underscores the potential for puppetry-based nutrition education to be integrated into broader health promotion and disease prevention programmes. Existing evidence from related fields, including oral health, sexual health, general health behaviours, and mental health promotion, suggests that puppets can facilitate dialogue, enhance recall of key messages, and support emotional processing among children and adults.^(2,8,17,55,56)^ Leveraging these constructs within well-designed, theoretically-based, and culturally tailored nutrition interventions may contribute to creatively addressing challenges in engaging diverse populations and translating nutrition knowledge into sustainable dietary change.

## Conclusion

This scoping review identified 25 nutrition education interventions that incorporated puppetry over nearly 45 years, indicating both a long-standing interest in utilising puppetry as an educational approach, and a relatively small, fragmented evidence base. Overall, the studies suggest that puppetry-based interventions support gains in nutrition knowledge and psychosocial engagement, whereas evidence for consistent effects on dietary behaviour and anthropometric outcomes remains limited and mixed. Puppet-based activities were typically implemented as part of multi-component interventions delivered to a wide range of populations and settings; while this approach reflects practical realities, it also complicates attribution of effects specifically to the use of puppetry in the intervention setting. There is a clear need for methodologically robust research studies that utilise standardised outcome measures, include longer follow-up time frames, and systematically report and provide assessment and evaluation on decisions about puppet type, character design, script development, cultural tailoring, and degree of audience participation. Such work would clarify when, for whom, and under what conditions puppetry-based nutrition interventions add unique value in improving nutrition knowledge as well as anthropometrics and dietary behaviour. Given the demonstrated ability to engage participants and to open dialogue around health topics in other domains, puppetry-based interventions remain a promising strategy to implement in nutrition education initiatives.

## Supporting information

10.1017/jns.2026.10102.sm001 Aboul-Enein et al. supplementary material Aboul-Enein et al. supplementary material

## Data Availability

All data generated and analysed during this review are included in the published review article.

## Supplementary material

The supplementary material for this article can be found at https://doi.org/10.1017/jns.2026.10102.
