# Supplementary material for: Incorporating the use of puppets and puppetry-based approaches in nutrition: a scoping review of interventions
Source: J Nutr Sci. 2026 May 12;15:e32. doi: 10.1017/jns.2026.10102 (PMC13168910; doi:10.1017/jns.2026.10102)
Supplement: Aboul-Enein et al. supplementary material [file S2048679026101025sup001.docx]

**Supplemental material.**

**Scopus database**

Nutrition [All Fields] OR Diet* [All Fields]

AND

“Intervention” [All Fields]; OR “Program” [All Fields]; OR “education” [All Fields]; OR “promotion” [All Fields]

AND

“Puppets” [All Fields]; OR “puppetry” [All Fields]; OR “puppeteer” [All Fields]; OR “puppet show” [All Fields]; OR “marionette” [All Fields]; OR “ventriloquy” [All Fields]; OR “ventriloquism” [All Fields]
